# A Novel End-to-End Biliary-to-Biliary Anastomosis Technique for Iatrogenic Bile Duct Injury of Strasberg-Bismuth E1-4 Treatment: A Retrospective Study and *in vivo* Assessment

**DOI:** 10.3389/fsurg.2021.747304

**Published:** 2021-10-28

**Authors:** Dong Ma, Pengpeng Liu, Jianwei Lan, Baiyang Chen, Yang Gu, Yun Li, Pengpeng Yue, Zhisu Liu, Deliang Guo

**Affiliations:** ^1^Department of Hepatobiliary Surgery, Zhongnan Hospital of Wuhan University, Wuhan, China; ^2^Department of Hepatobiliary Surgery, Affiliated Jingmen First People's Hospital of Hubei University for Nationalities, Jingmen, China

**Keywords:** iatrogenic bile duct injury, anastomosis technique, bile duct repair, postoperative benign biliary stricture, biliary reconstruction

## Abstract

**Background:** An iatrogenic bile duct injury (IBDI) is a severe complication that has a great impact on the physical and mental quality of life of the patients, especially for patients with postoperative benign biliary stricture. The effective measures for end-to-end biliary-to-biliary anastomosis intraoperative are essential to prevent the postoperative bile duct stricture, but also a challenge even to the most skilled biliary tract surgeon.

**Objective:** A postoperative benign biliary stricture is an extremely intractable complication that occurs following IBDI. This study aimed to introduce a novel end-to-end biliary-to-biliary anastomosis technique named fish-mouth-shaped (FMS) end-to-end biliary-to-biliary reconstruction and determine the safety and effectiveness for preventing the postoperative benign biliary stricture in both rats and humans.

**Methods:** In this study, 18 patients with biliary injury who underwent an FMS reconstruction procedure were retrospectively analyzed. Their general information, disease of the first hospitalization, operation method, and classification of bile duct injury (BDI) were collected. The postoperative complications were evaluated immediately perioperatively and the long-term complications were followed up at the later period of at least 5 years. An IBDI animal model using 18 male rats was developed for animal-based evaluations. A bile duct diathermy injury model was used to mimic BDI. The FMS group underwent an FMS reconstruction procedure while the control group underwent common end-to-end biliary-to-biliary anastomosis, a sham operation group was also established. The blood samples, liver, spleen, and common bile duct tissues were harvested for further assessments.

**Results:** In the retrospective study, there was no postoperative mortality and no patient developed cholangitis during the 5-years postoperation follow-up. In the study of IBDI animal models, compared with the control group, the FMS reconstruction procedure reduced the occurrence of benign biliary stenosis, liver function damage, and jaundice. The blood tests as well as morphological and pathological observations revealed that rats in the FMS reconstruction group had a better recovery than those in the control group.

**Conclusions:** An FMS reconstruction procedure is a safe and efficient BDI treatment method.

## Introduction

An iatrogenic bile duct injury (IBDI) mainly occurs as a severe complication of laparoscopic cholecystectomy (1, 2). It is a potentially life-threatening condition and is characterized by high morbidity ranging from 2.3 to 23% and mortality from 0.07 to 0.17% (3). Cognizant of this, it is a condition that has a great impact on the physical and mental quality of life of the patients (2–4). This is especially the case for patients diagnosed with postoperative benign biliary stricture (5–7). Misidentification of the common bile duct or an aberrant hepatic duct as the cystic duct, inappropriate clipping, and thermal damage represents the most frequent cause of IBDI following cholecystectomy (8), and the incidence of IBDI following cholecystectomy is 0.32–0.52% (7). There is also a considerable risk of IBDI occurring even when intraoperative cholangiography is used (9).

Early diagnosis and proper treatment of IBDI is important for the patients because ineffective measures may lead to bile duct stricture and subsequent serious complications, such as recurrent cholangitis, hepatic failure, and even death (5, 10, 11). The preoperative accurate assessment of IBDI is the basis of making a reasonable treatment strategy as well as selecting the appropriate operation mode to ensure the success of the operation (12). The types of IBDI are various and complex. Nowadays, the Strasberg–Bismuth classification system is recommended by several guidelines or standards (13–15). There is relatively no dispute about the surgical scheme of biliary reconstruction for patients with A–E five types of Strasberg-Bismuth (16). However, there are controversial issues in gastrointestinal surgery regarding biliary reconstruction methods for the patients with IBDI diagnosed with Strasberg-Bismuth E1–4. These cases are patients with proximal location biliary injury and in which hepatic junction is preserved (type E1–E2), or with more proximal injuries (type E3–E4) in which the integrity of the main hepatic duct junction deteriorated. The different biliary reconstructions have been reported in these IBDI types (Strasberg-Bismuth E1-4): Roux-en-Y hepaticojejunostomy (HJ), end-to-end ductal anastomosis, Lahey HJ, jejunal interposition hepaticoduodenostomy, Blumgart (Hepp) anastomosis, Heinecke-Mikulicz biliary plastic reconstruction, and Smith mucosal graft (17). Currently, Roux-en-Y HJ is the most common biliary reconstruction method in these surgical schemes, owing to its lower number of postoperative anastomosis strictures with HJ than with end-to-end ductal anastomosis (18). However, Roux-en-Y HJ is not physiologic, and is associated with different disturbances in the release of gastrointestinal hormones leading to maldigestion and malabsorption. In recent years, the traditional method of HJ has been challenged by end-to-end biliary reconstruction in these groups of patients (19). The achievement of good long-term results is possible in the patients undergoing end-to-end ductal anastomosis, this is because it is more physiologic than HJ and it is associated with fewer early postoperative complications. Herein, we introduce a new procedure of fish-mouth-shaped (FMS) end-to-end biliary reconstruction and evaluate its safety and long-term efficacy in the patients. Furthermore, the analyses of why FMS end-to-end ductal anastomosis is better than the traditional anastomosis were done using an IBDI rat model.

## Materials and Methods

### Use of Human Subjects—Ethic Statement

When inviting the participants, the purpose of the research was clearly explained. Meanwhile, written informed consent was given by all the participating patients or their legal representatives. Before the inclusion of the first individual, the ethics committee approval was obtained from the local ethics committee of the Affiliated Jingmen First People's Hospital of Hubei University for Nationalities, Hubei, China, in accordance with the Declaration of Helsinki.

### Use of Experimental Animals—Ethic Statement

All animal experiments were approved by the Wuhan University Animal Welfare and Ethical Review Body (AWERB) and the study was performed under a license issued by the home office. All animal care and experimental procedures were in accordance with the Roche guidelines and the national/international guidelines for animal care.

### Bile Duct Injury (BDI), Reconstruction, and Quantification of Postoperative Biliary Stricture and Organ Dysfunction

**Figure 1:**
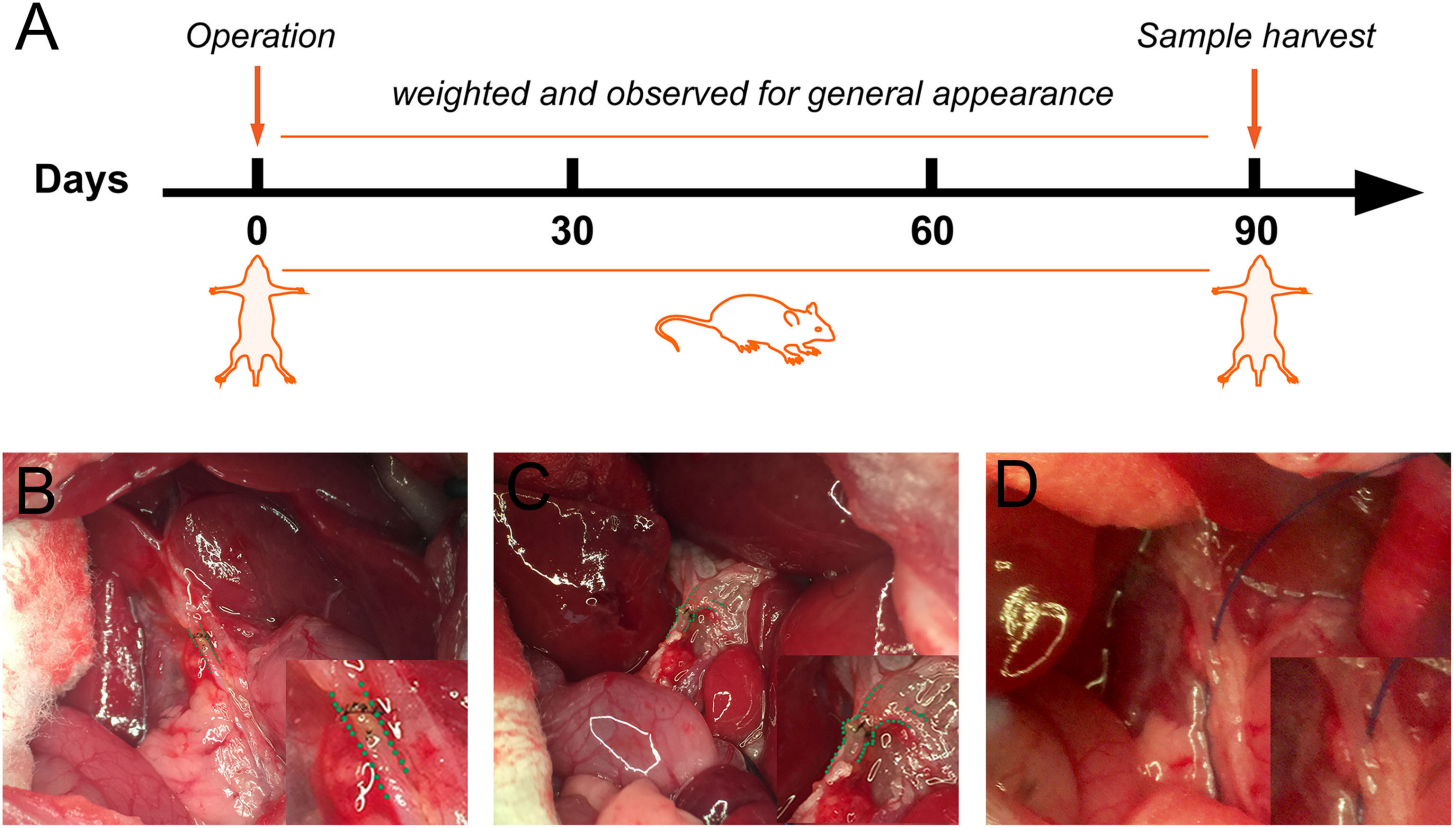
Schematic representation of the experimental procedure and anastomosis methods for bile duct injured rats. **(A)** The flowchart of rat experiment. **(B)** Traditional biliary anastomosis in the control group. Common bile duct after an end-to-end suture was highlighted by a green dashed line. **(C)** “Fish-mouth to fish mouth” anastomosis was done in the fish mouth shaped (FMS) group. The highlighted common bile duct was reconstructed by a wider anastomosis. **(D)** The common bile duct was isolated in the sham group.

The present study was carried out on 18 male Sprague-Dawley (SD) rats (Charles River Ltd, Pecking, China) weighing from 250 to 300 g receiving a standard diet and water ad libitum. They were randomized into three treatment groups: (1) the sham-operated rats (sham group) treated with laparotomy and bile duct isolated, (2) the traditional end-to-end anastomosed rats (control group) underwent laparotomy, bile duct transected, and traditional end-to-end anastomosis using a 10–0 nylon suture. (3) the FMS reconstructed rats (FMS group) underwent laparotomy, bile duct transected, and FMS reconstruction using a 10–0 nylon suture. The follow-up examinations were performed in the third postoperative month, and the blood samples, liver, spleen, and common bile duct tissues were harvested for further assessments. The timeline of the experimental procedures was presented in Figure 1A.

### Patient Population

We retrospectively analyzed the results of 18 patients with biliary injury who underwent an FMS reconstruction procedure in Affiliated Jingmen First People's Hospital of Hubei University for Nationalities between June 2007 and August 2015. Their general information, disease of the first hospitalization, operation method, and classification of bile duct injury (BDI) were collected. The postoperative complications were evaluated immediately perioperatively (within 2 weeks to detect the short-term complications) and at the later period of atleast 5-year follow-up (to detect the long-term complications).

### FMS Reconstruction Procedure

#### For Patients

(1) The degree of BDI was first evaluated, the patients whose bile ducts had been transected or semi-transected were allowed to the following procedure, even if they had a defective partial bile duct. (2) The tension after bile duct reconstruction was then pre-estimated. In this procedure, three strategies were recommended to attempt to keep a tension-free anastomotic stoma: dissecting and descending the hilar plate, freeing the liver by cutting the peripheral ligaments and freeing the pancreaticoduodenal en-block by Kocher maneuver. (3) The devitalized tissues were then, pruned and the two ends of the bile duct were reshaped into a “fish-mouth.” (4) A continuous 6–0 absorbable polydioxanone sulfate was then used to finish the “fish-mouth” to “fish-mouth” anastomosis. (5) T-tube drainage was used away from the anastomose ostium. An abdominal cavity drainage tube was placed *via* the foramen of Winslow below the hepatoduodenal ligament. It is important to note that the integrity of the main hepatic duct junction deteriorated for E4 injury, we first reconstructed the left hepatic duct and the right hepatic duct, and then, FMS end-to-end biliary-to-biliary reconstruction was performed. The key steps of bile duct reshaping and reconstruction were illustrated in the Supplementary Flash Videos.

#### For Rats

(1) The FMS reconstruction procedures in rats were performed under a dissecting microscope. (2) The rats were first anesthetized using 10% chloral hydrate. In the two experimental groups, the common bile ducts (approximately 3 mm distal to the biliary confluence) were injured by electrical coagulation for 0.5 s with bipolar forceps connected to a generator (SPRING CHR-V Bipolar Coagulator; SPRING Medical Products, Wuhan, China). The coagulative bile duct tissue was dissected and removed. The BDI was established. (3) An end-to-end anastomose was then performed using a 10–0 nylon suture in the control group. (4) In the FMS group, both ends of the bile duct were first reshaped into a “fish-mouth” before an end-to-end anastomose. (5) The abdominal cavity was then closed and sutured, and the rats were placed on a warm stage to promote walking. Representative photographs in the three groups after the intraoperative key step are shown in Figures 1B–D.

### Blood Test

The complete blood count and liver function tests were done before the operation and during the periodic outpatient follow-up examination. The liver function test in rats was performed at the end of the study in the third post-operative month.

### H and E Staining and Masson Staining

Briefly, the tissues were obtained, fixed, and processed to obtain 4 μm sections. The slides were deparaffinized, rehydrated, and stained with H&E (Sigma, USA) by a standard protocol. We visualized the whole image of the pathological liver and evaluated the infiltration of neutrophils and eosinophils to predict liver inflammation. Masson staining was used for the detection of hepatic fibrosis. The deparaffinized and rehydrated slides were incubated with a Masson staining mixture, stained with a phosphomolybdic acid-aniline blue solution by a standard protocol. The collagen volume fraction (CVF) was calculated by Image Pro-Plus 6.0.

### Immunohistochemistry

After deparaffinization, dehydration, and antigen retrieval, endogenous peroxidase activity block was immersed in 3% hydrogen peroxide and then 10% goat serum for 1 h. The liver tissue slides were then incubated overnight at 4°C with the primary antibodies against α-smooth muscle actin (α-SMA, 1:500; Cat# 23081-1-AP; Proteintech, Wuhan, China, 1 h, room temperature), and the bile duct slides were deal with CK19 antibody (1:100; Cat# A19015; ABclonal, Wuhan, China, 1 h, room temperature) using the same program. Biotin-conjugated Affinipure Goat Anti-Rabbit IgG(H+L) (CAT# SA00004-2 Proteintech) was applied for 60 min at room temperature. The slides were later stained with 3,3-diaminobenzidine (DAB) at room temperature, lightly counterstained with hematoxylin, dehydrated, and glass cover slips were covered. The quantification of immunoreactivity was performed using Image Pro-Plus 6.0, and 3–5 fields were randomly selected from each slide to determine the positive areas related to the total view. All the quantifications were performed in a blind manner.

### Follow-Up

Follow-up of all the patients was done through telephone inquiries or special clinic re-examinations for at least 5 years. The date of the surgery was defined as the initiation time. The special clinic re-examinations involved laboratory tests, such as routine blood tests, liver function tests, and alkaline phosphatase (ALP) level tests. MRI or CT scans of the abdomen were also done after the treatment. They were done once in every 6–12 months for the first year and then once in every year for at least 5 years unless death occurred earlier.

### Statistics

Data are presented as mean value ± SD for the continuous variables and proportions for the categorical variables. The continuous variables were analyzed by the Student's *t*-test and the categorical variables were analyzed using the chi-squared test or Fisher's exact test, respectively. GraphPad Prism 6.0 (CA, USA) was used for the statistical analysis and plotting. For all comparisons, the statistical significance was accepted when *p* < 0.05.

## Result

### The Surgical Characteristics and Outcomes of the Patients

**Figure 2:**
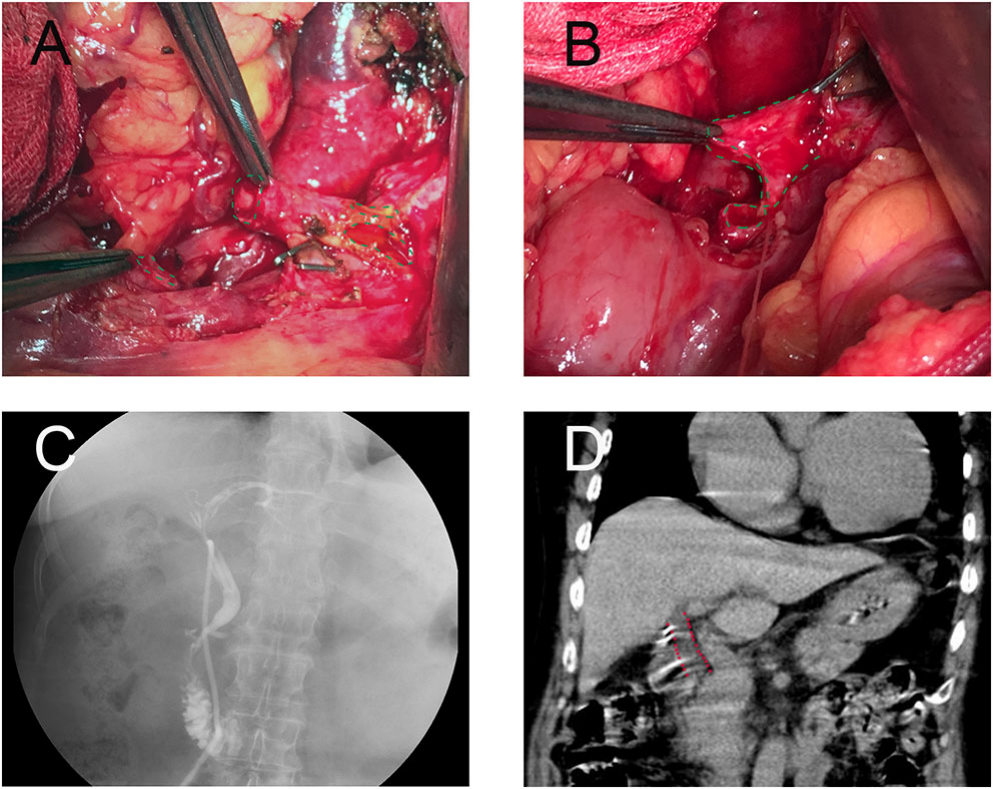
A patient with type E2 combined with type D (Strasberg-Bismuth) bile duct injury (BDI) underwent FMS end-to-end biliary-to-biliary reconstruction. **(A)** Intraoperative photograph of bile duct before shaping, bile duct gaps were represented by a green dashed line. **(B)** Intraoperative photograph of bile duct after fish mouth shaping, the bile duct was reshaped as a fish-mouth. **(C)** T-tube cholangiography before T-tube removed. **(D)** CT-scan at year 2 postoperative. The common bile duct was highlighted (red dashed line), no stenosis was found.

The patients comprised of seven male patients (41.1%) and 11 females aged between 25 and 72 years at the time of surgery (Table 1). All the patients had been diagnosed with acute cholecystitis complicated with gallstones. Two had been diagnosed with acute suppurative cholecystitis, one with acute suppurative cholecystitis with Mirizzi syndrome, and one with gangrenous cholecystitis. During the bile duct reconstruction operation, BDI was diagnosed, typed, and classified according to the BDI classification of Strasberg-Bismuth. Six (33.3%) patients were diagnosed with type E1 BDI, seven (38.9%) with type E2, four (22.2%) with type E3, one (5.6%) with a combination of types E4 and E2. The intraoperative photographs and postoperative examination photographs of a patient diagnosed with a combination of types E4 and E2 BDI underwent FMS end-to-end biliary reconstruction are shown in Figure 2. Most patients (72.2%) have a mild bile leakage duration of between 0 and 3 days after operation, five (27.8%) patients experienced bile leakage for more than 3 days. For the five, the bile leakage was ranged between 20 and 200 ml per day for 3–8 days. There were no reoperations. Only two patients had mild postoperative abdominal pain while none had a fever. Moreover, there was no postoperative mortality. During the follow-up examinations, no patient developed cholangitis. All the patients had normal liver function except one with an unremarkable increased ALP. Follow-up CT or MRI tests did not reveal any signs of cholangitis nor benign biliary stricture.

**Table 1:** Demographics and clinical characteristics.

**Variables**	**N = 18**
**Demographics**	
Age, mean(SD)	49.9 (13.6)
Sex, n (%)	
Male	7 (38.9%)
Female	11 (61.1%)
Diabetes mellitus, n (%)	
Yes	1 (5.6%)
No	17 (94.4%)
Hypertension, n (%)	
yes	2 (11.1%)
No	16 (88.9%)
**Indications and preoperative status**	
Indication of cholecystectomy, n (%)	
Acute cholecystitis with gallstone	14 (77.8%)
Acute suppurative cholecystitis with gallstone	2 (11.1%)
Gangrenous cholecystitis with gallstone	1 (5.6%)
Acute suppurative cholecystitis with Mirizzi syndrome	1 (5.6%)
Initial type of operation	
LC	16 (88.9%)
OC	2 (11.1%)
Conversion to open	
No	None
Yes	16 (100%)
**Strasberg-Bismuth classification of BDI**	
E1	6 (33.3%)
E2	7 (38.9%)
E3	4 (22.2%)
E4 + E2	1 (5.6%)
**Procedure specific complications**	
Perioperative mortality	none
Biliary fistula/leak days	
0–3	13 (72.2%)
3–6	3 (16.7%)
6–8	2 (11.1%)
Bile duct stenosis	none

*SD indicates standard deviation; LC, laparoscopic cholecystectomy; OC, open cholecystectomy; BDI, bile duct injury*.

### FMS Reconstruction Reduced the Occurrence of Benign Biliary Stricture *in vivo*

**Figure 3:**
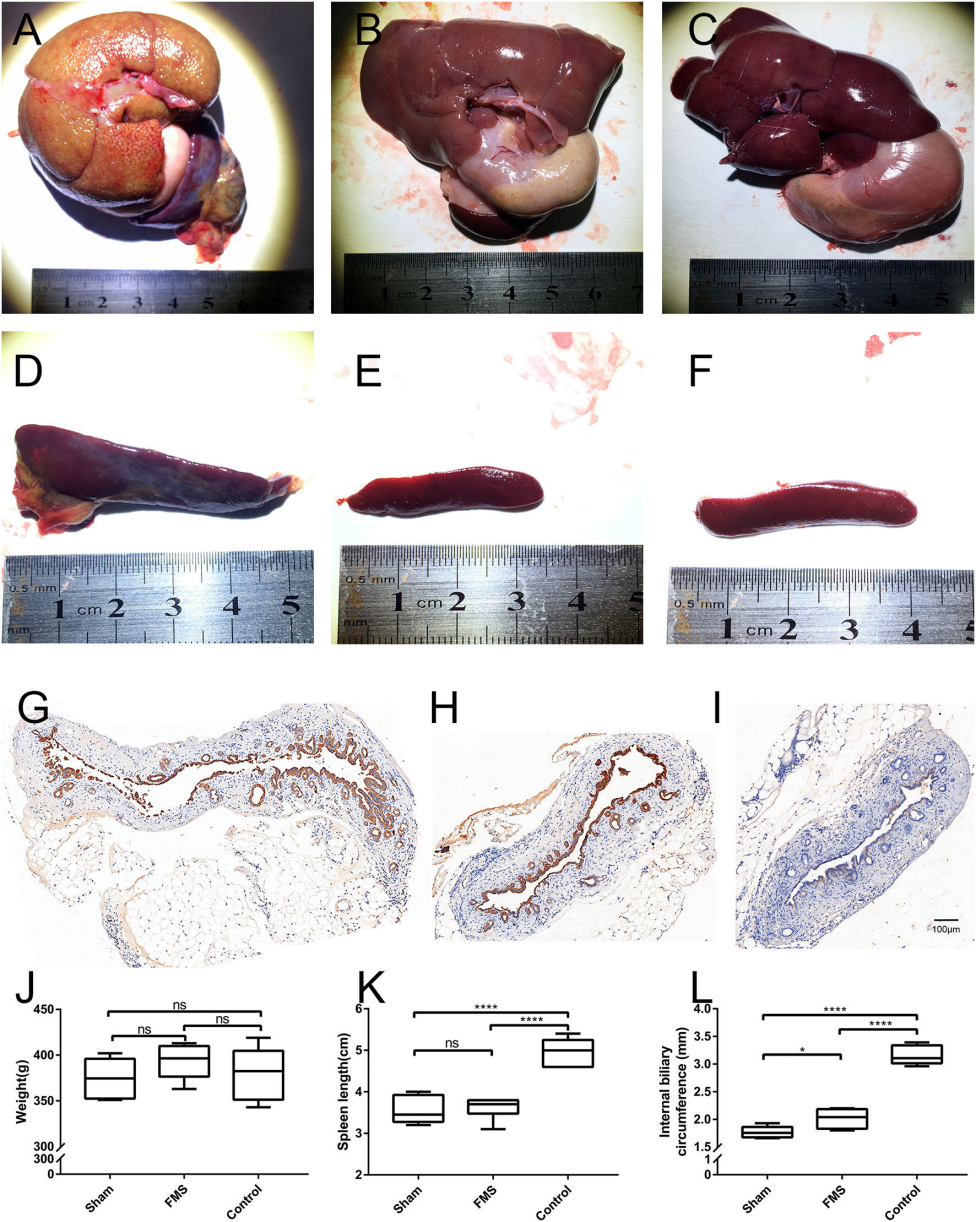
The FMS reconstruction relieves the incidence of benign biliary stricture *in vivo*. The liver, spleen, and proximal bile duct were obtained after the rats were sacrificed. The liver in the control group **(A)** showed visible cholestasis, jaundice compared with the FMS group **(B)** and sham group **(C)**. Splenomegaly was found in the control group **(D)**, however, no significant differences were seen in the FMS group **(E)** and sham group **(F)**. The length of the spleen was compared **(K)**, the FMS reconstruction could relieve splenomegaly compared with the traditional anastomosis method. The proximal duct was transversely sliced and stained with a CK19 antibody. The internal biliary circumference was measured using Aperio ImageScope **(L)**. A significantly dilated bile duct was identified in the control group **(G)**. there is only mild dilatation of the bile duct in the FMS group **(H)** compared with the control group **(I)**. ****P < 0.0001.

The efficacy of FMS reconstruction in rats was assessed 3 months after the operation. Because the bile duct is a deficiency of structural rigidity, the circumference of the inner wall of the proximal common bile duct was used to evaluate the anastomotic stenosis and patency. The postoperative circumference of the inner wall in the FMS reconstruction group was smaller than that of the control group. However, akin to that of the sham group (Figure 3), the proximal extrahepatic bile ducts were dilated in the control group but not dilated in the FMS group. Additionally, dilated bile ducts are regularly seen as a sign of cholestasis. Obvious hepatomegaly, splenomegaly, ascites (data not shown), gastric, and omental varices were found in the control group. On the contrary, this was not observed in the FMS and sham groups. Besides those, the liver in the control group also showed visible cholestasis, jaundice, and a hard texture. However, this was not the case in the FMS and sham groups. The two groups had no significant differences in the cholestasis, jaundice, and texture of the livers.

### FMS Reconstruction Relieves the Incidence of Liver Injury *in vivo*

**Figure 4:**
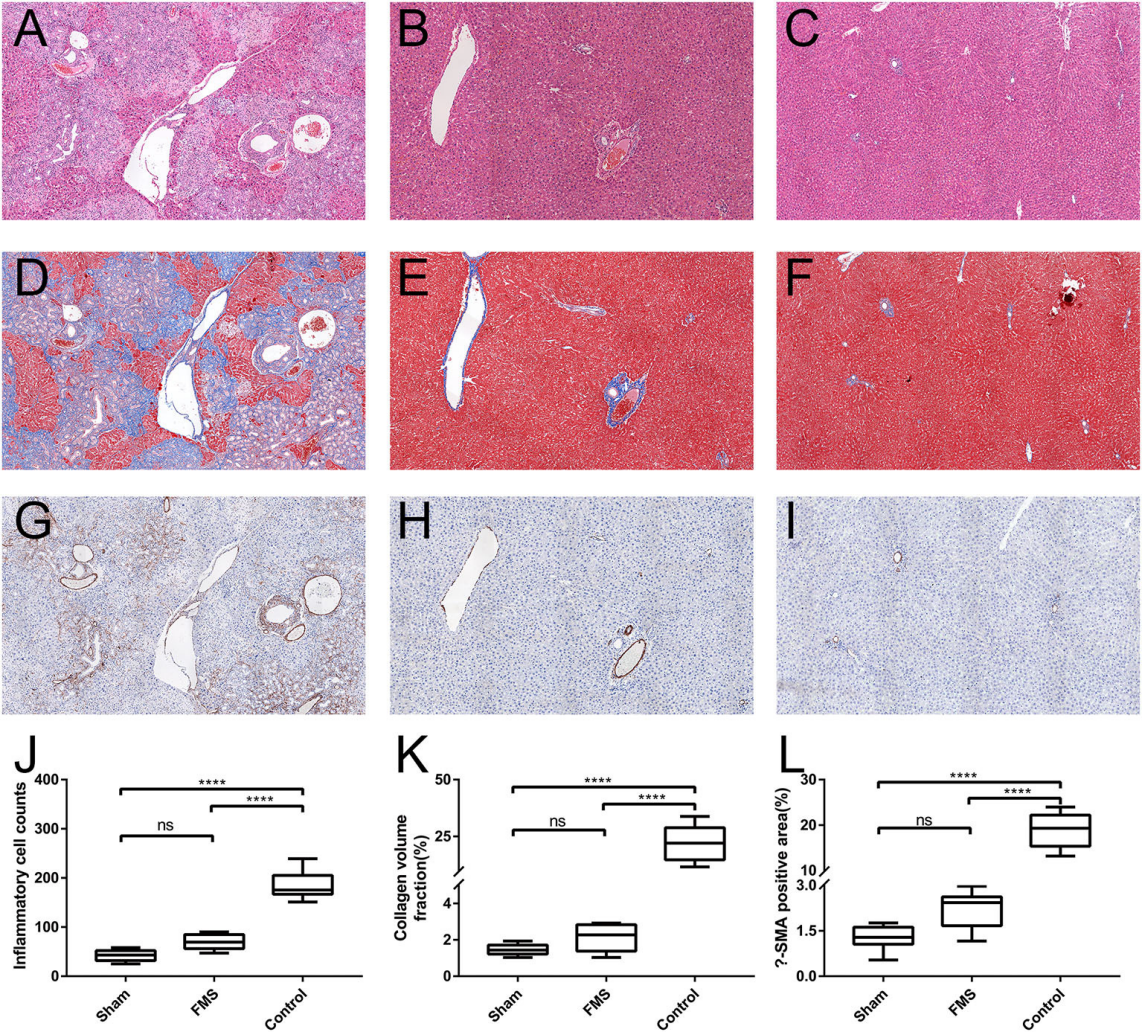
The FMS reconstruction relieves the incidence of liver injury *in vivo*. The sections of liver tissue were stained with H&E. [**(A)** control group. **(B)** FMS group. **(C)** sham group], Masson [**(D)** control group. **(E)** FMS group. **(F)** sham group] and α-smooth muscle actin (α-SMA) antibody [**(G)** control group. **(H)** FMS group. **(I)** sham group]. The interstitial inflammatory cells were calculated in each group and measured **(J)**. There were lots of inflammatory cell infiltrations in the control group, but insignificant differences between the FMS group and sham group. Collagen volume fraction (CVF) was evaluated using Image Pro-Plus 6.0, there was no statistically significant difference in the FMS group and control group **(K)**. The α-SMA positive area in the three groups was also evaluated, similar results were seen with CVF **(L)**. ****P < 0.0001.

The results of the pathological section are consistent with the morphological observations (Figure 4). In the control group, the pathological sections of the liver of rat showed distended capillary bile ducts infiltrating with inflammatory cells. However, in the FMS group, only gentle inflammatory cells were seen compared with the sham group. Further to this, Masson staining and α-SMA immunohistochemical staining used to evaluate liver and bile duct fiber hyperplasia revealed the least amount of overall distribution of collagen in the sham and FMS sections, but extensive in the control group. In accordance with the Masson staining results, except for the control group, no remarkable expression of α- SMA was observed in the sham and FMS groups.

### FMS Reconstruction Attenuated Bile Duct Anastomosis-Induced Liver Damage

**Figure 5:**
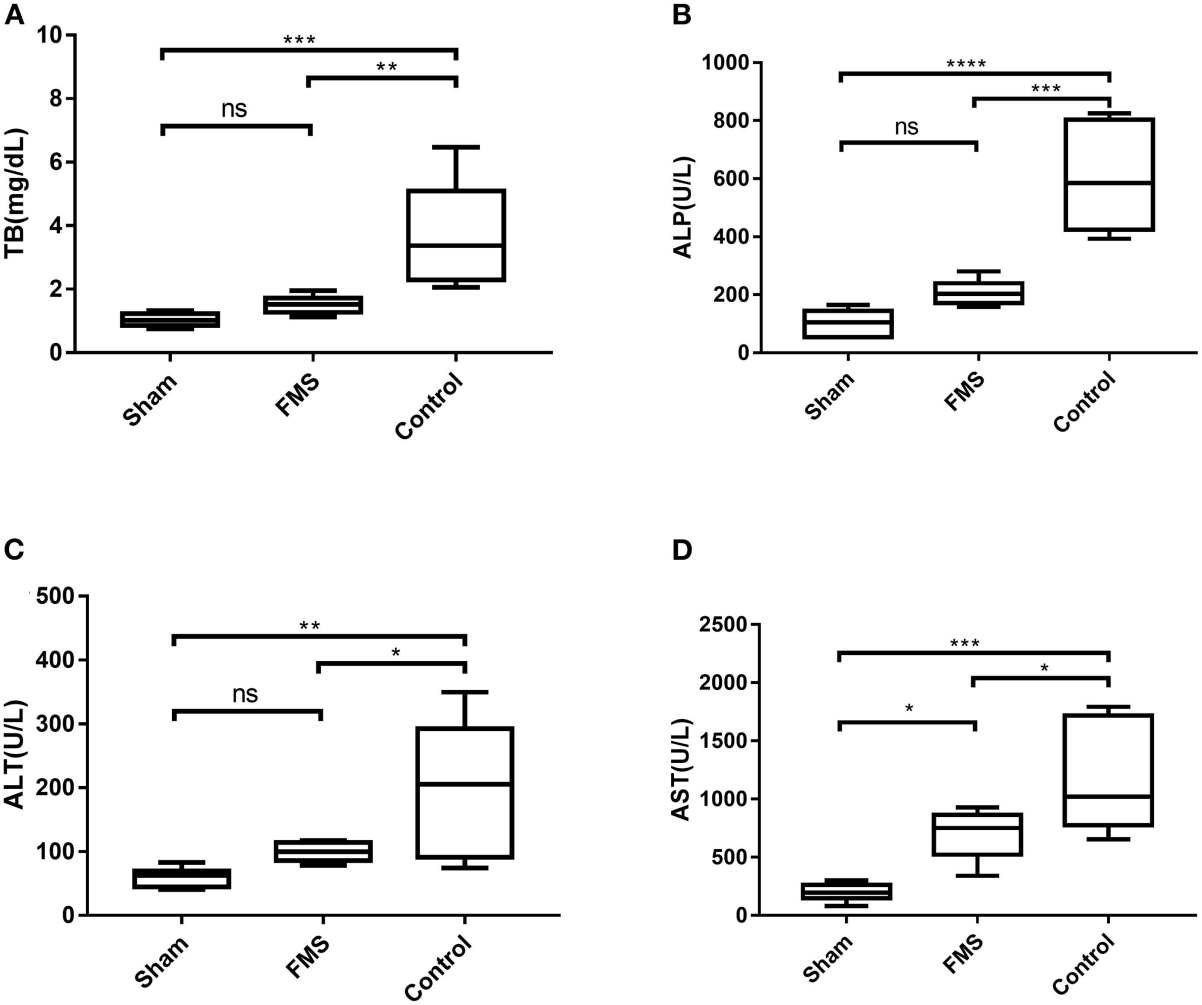
The FMS reconstruction attenuates bile duct anastomosis-induced liver damage. **(A)** Total bilirubin (TB) of the three groups. **(B)** alkaline phosphatase (ALP) of the three groups. **(C)** ALP of the three groups. **(D)** AST of the three groups. *P < 0.05, **P < 0.01, ***P < 0.001, and ****P < 0.0001.

The liver function tests were done while the rats were sacrificed for tissue harvest (Figure 5). The total bilirubin (TB) in the control group was higher than in the FMS group and sham group. However, there was no significant difference between the TB in the FMS group and the sham group. Similar results were obtained in the Aspartate aminotransferase (AST) and Alanine aminotransferase (ALT) tests. However, the FMS group had a mild increase in AST (U/L, 699.09 ± 211.23 vs. 199.02 ± 76.39, *p* = 0.020) and the ALT levels (U/L, 99.24 ± 15.09 vs. 59.66 ± 15.14, *p* = 0.533) compared with the sham group. The ALP tests were also done because it is more sensitive in obstructive liver injury. Both the FMS group and control group had increased ALP levels. However, the ALP levels in the control group were three times higher than those of the FMS group. These results indicated that FMS reconstruction had the potential to attenuate the end-to-end anastomosis-induced liver damage.

## Discussion

Early diagnosis and proper treatment of IBDI is important to prevent bile duct stricture and other subsequent serious complications, such as recurrent cholangitis, a hepatic failure that can lead to death (20). Endoscopic therapy (21, 22) or covered stent placement (23) have been suggested as desirable treatments for mild BDI, especially those sidewall damages with a continuous bile duct structure. However, these treatment options were not explored in this study. An interrupted suture repair (23) can be made when a small tear is noted, and this kind of BDI is not discussed in this paper. The surgical treatments are performed to restore the biliary-enteric continuity (24). The reconstruction procedures, such as duct-to-duct biliary reconstruction and hepaticojejunostomy, are the major contributors to patients diagnosed with transection injuries.

Herein, we introduce a strategy of an end-to-end suture method suitable for biliary transection injury, especially for the types of Strasberg-Bismuth E1–4. This reconstruction approach has several advantages over conventional procedures. First, it completely preserves the normal functioning of the sphincter of Oddi, and it is closer to physiologic states. Second, reshaping the ends of the bile duct into a fish mouth shape (FMS) makes it easier to anastomose because a wider stoma is established. Moreover, the wider anastomosis can relieve subsequent benign biliary stricture. Meanwhile, we have several tips that ensure the success of this procedure: (1) to control the tension, this procedure is limited to the patients whose biliary defect length is <2–2.5 cm. (2) Dissecting and descending the hilar plate (25), freeing the liver by cutting peripheral ligaments, freeing the pancreaticoduodenal en-block by using the Kocher maneuver, tracting, and then suturing of the upper edge of the pancreas and the soft tissue of porta hepatis are strategies further suggested to make it easier to finish the procedure. (3) A T-tube is recommended to brace the bile duct for at least 6 months (26). (4) The integrity of the main hepatic duct junction deteriorated for E4 injury, we first reconstructed the left hepatic duct and the right hepatic duct, and then FMS end-to-end biliary-to-biliary reconstruction was performed.

We conducted a retrospective study of 18 patients with BDI who underwent FMS reconstruction. All the patients were placed on a follow-up program for at least 5 years, T-tube was removed at 6–8 months after the operation. In addition, CT or MRI tests were performed periodically during the 5 years postoperation follow-up period. No serious complications nor deaths were reported during the perioperative period. Only mild to moderate biliary leakage occurred, with a recovery period no longer than 8 days. No patient developed cholangitis in the follow-up period. The CT examination showed a direct unobstructed biliary drainage even in the patient initially diagnosed with a combination of IBDI types. Based on the results of this study, this anastomosis method was found to be safe and effective in the IBDI treatment. Herein, all of the 18 patients recovered well. The results may be attributed to the ability of the larger caliber anastomosis to relieve anastomotic stenosis caused by local inflammation. There are, however, other possible explanations. Different from homogenous anastomosis tension caused by traditional anastomosis, a fish-mouth to fish-mouth anastomosis could make a local partial low tension, which can make a better recovery to the bile duct.

To evaluate the effect of this anastomosis, a BDI animal model was developed (27). The common bile duct was electrically injured and transected to mimic that of the patients with BDI. It is encouraging to see that the FMS reconstruction group had a better performance compared with the control group. In the FMS group, the inner wall perimeter of the proximal common bile duct, liver function damage, jaundice was restored, and only an insignificant increase of liver enzymes was confirmed. Cognizant of this, this animal model reinforces our conclusion discovered in the retrospective study despite the model being unable to simulate specific situations, such as underlying diseases, degree of local inflammation, and blood supply of injured bile duct.

However, human IBDI is more complex and diversified. It is difficult to establish an ideal animal model completely imitating the human IBDI. To the best of the authors' knowledge, there are no relatively mature protocols to establish a diathermy BDI model. The diathermy injuries are hard to evaluate. Even in the clinical evaluation and treatment of BDI, the severity score of diathermy injuries is rarely included in most classification criteria of systems. Meanwhile, this study was limited by the small number of enrolled patients and thus the findings could be skewed to some extent. Moreover, the study did not explore other treatment methods to conclusively make comparisons. As such, future multi-centered prospective studies should be undertaken to investigate these effects.

In this study, we do, however, find a new procedure of FMS end-to-end biliary reconstruction more proper to reconstruct BDI during operation, especially for biliary transection conditions. Evidently, the FMS reconstruction procedure can prevent bile duct stricture safely and effectively. This procedure should help others to handle a new strategy when BDI occurs.

## Data Availability Statement

The raw data supporting the conclusions of this article will be made available by the authors, without undue reservation.

## Ethics Statement

The studies involving human participants were reviewed and approved by Ethics Committee of Affiliated Jingmen First People's Hospital of Hubei University for Nationalities. The patients/participants provided their written informed consent to participate in this study. The animal study was reviewed and approved by Wuhan University Animal Welfare and Ethical Review Body (AWERB). Written informed consent was obtained from the individual(s) for the publication of any potentially identifiable images or data included in this article.

## Author Contributions

DG, DM, and ZL: conception and design. DG, DM, ZL, and PL: development of methodology. DG, DM, YG, YL, and ZL: acquisition of data (provided animals, acquired and managed patients, provided facilities, etc.). PL and BC: analysis and interpretation of data (e.g., statistical analysis, biostatistics, and computational analysis). DM, PY, ZL, and DG: writing, review, and/or revision of the manuscript and administrative, technical, or material support (i.e., reporting or organizing data and constructing databases). ZL and DG: study supervision. All authors contributed to the article and approved the submitted version.

## Funding

This work was supported by grants from the National Natural Science Foundation of China (No. 81772926 to ZL), the Zhongnan Hospital of Wuhan University Science, Techonology and Innovation Seed Fund (CXPY2020015), and the Cancer research and translational platform project of Zhongnan Hospital of Wuhan University (ZLYNXM202004).

## Conflict of Interest

The authors declare that the research was conducted in the absence of any commercial or financial relationships that could be construed as a potential conflict of interest.

## Publisher's Note

All claims expressed in this article are solely those of the authors and do not necessarily represent those of their affiliated organizations, or those of the publisher, the editors and the reviewers. Any product that may be evaluated in this article, or claim that may be made by its manufacturer, is not guaranteed or endorsed by the publisher.
